# LocusTrack: Integrated visualization of GWAS results and genomic annotation

**DOI:** 10.1186/s13029-015-0032-8

**Published:** 2015-02-03

**Authors:** Gabriel Cuellar-Partida, Miguel E Renteria, Stuart MacGregor

**Affiliations:** Statistical Genetics, QIMR Berghofer Medical Research Institute, Brisbane, Australia; School of Medicine, University of Queensland, St. Lucia, Queensland Australia; Genetic Epidemiology, QIMR Berghofer Medical Research Institute, Brisbane, Australia

## Abstract

**Background:**

Genome-wide association studies (GWAS) are an important tool for the mapping of complex traits and diseases. Visual inspection of genomic annotations may be used to generate insights into the biological mechanisms underlying GWAS-identified loci.

**Results:**

We developed LocusTrack, a web-based application that annotates and creates plots of regional GWAS results and incorporates user-specified tracks that display annotations such as linkage disequilibrium (LD), phylogenetic conservation, chromatin state, and other genomic and regulatory elements. Currently, LocusTrack can integrate annotation tracks from the UCSC genome-browser as well as from any tracks provided by the user.

**Conclusion:**

LocusTrack is an easy-to-use application and can be accessed at the following URL: http://gump.qimr.edu.au/general/gabrieC/LocusTrack/. Users can upload and manage GWAS results and select from and/or provide annotation tracks using simple and intuitive menus. LocusTrack scripts and associated data can be downloaded from the website and run locally.

## Background

Genome-wide association studies (GWAS) have revolutionised the genetic mapping of complex traits and diseases over the last decade [1-3]. However, a considerable amount of the markers identified to date lie within non-coding regions and/or might be only proxy markers to the actual causal variants [2,3]. Tools that aid the visual inspection of these loci may facilitate the identification of functional elements located near GWAS-associated variants. LocusZoom [4] and SNAP-plot [5] have become widely used tools to generate locus-specific graphical displays of association results in the context of linkage disequilibrium (LD) as well as the position relative to nearby genes and local recombination hotspots. However, it is now becoming increasingly important to also visualise GWAS results in the context of functional annotations beyond genes (e.g. chromatin state, transcription factor binding sites, phylogenetic conservation, etc.) [6]. Thus, we have developed LocusTrack, a web-based application that allows the user to generate regional GWAS results plots that incorporate genomic annotations within the same figure. Currently LocusTrack supports both user-provided custom tracks as well as tracks from the UCSC genome-browser.

### Implementation

#### Features and functionality

**Figure 1:**
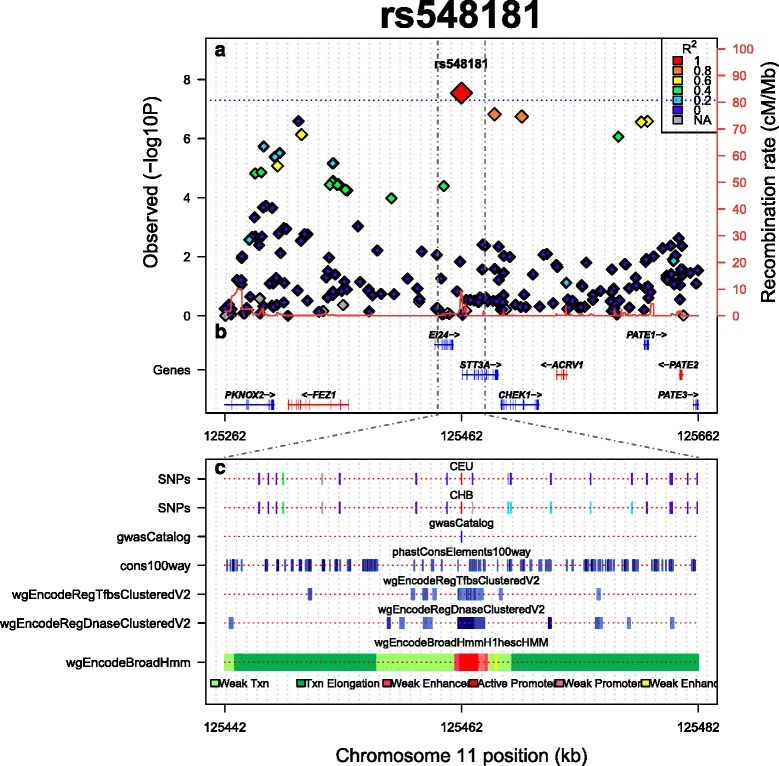
**Example of a LocusTrack plot.** The plot shows regional GWAS results **(a)** from publicly available GWAS results of the schizophrenia working group along with the genes within the region **(b)** and seven genomic annotation tracks **(c)**.

LocusTrack plots display regional GWAS results in the top panel (Figure 1a). Here, the user can opt between showing *P*-values on the –log10 scale (i.e. LocusZoom-like fashion) on the left y-axis or displaying LD (*r*^2^) (i.e. SNAP-like fashion), which is often useful for investigating a region in the absence of *P*-values. Recombination rates are represented on the right y-axis. By default, LocusTrack selects the SNP with the strongest association and generates a plot according to a user-defined window-frame size. However, it is also possible for the user to specify any other SNP(s) if desired. The plot also shows the pairwise (LD) pattern of each SNP with the user specified SNP. Users can choose to compute LD (*r*^2^) estimates from different 1000 Genomes Project populations available.

The second LocusTrack panel displays symbol and location of genes within that region (Figure 1b). Intron and exon positions are displayed in a similar fashion to LocusZoom. Orientation of the transcribed strand is indicated by differential colouring (blue = plus strand; red = minus strand) and arrows. The position of gene symbols is automatically adjusted to minimise the area occupied in the figure and to avoid overlap with one another.

LocusZoom provides the option for the data point to reflect different genomic annotations such as synonymous variants, splice variants, transcription factor binding sites, conservation, and whether they are in the GWAS catalogue. LocusTrack can incorporate any type of annotation in the form of genomic tracks in a third panel (Figure 1c). In this way, the user can specify between 1 and 10 different tracks which can be either custom tracks (i.e. the user must upload the data), LD tracks (i.e. a track displaying LD of the SNPs in another population), or publicly-available UCSC tracks. Note that LocusTrack uses the bioconductor package rtracklayer [7] to retrieve and parse UCSC tables. However, some tables come in a non-parseable form, or are truncated by the UCSC browser if they exceed certain limits (usually around 100,000 records), so they cannot be obtained by the program. This is particularly true for *wiggle* and *big-wiggle* format files. However, for these cases, the user can download directly the tracks via UCSC Table browser (http://genome.ucsc.edu/cgi-bin/hgTables) and input them as custom tracks.

Our application also allows users to zoom in and focus on a smaller region in the bottom panel, drawn from that shown in the first two panels. This provides a closer look to the annotation tracks at the region of interest, without modifying the plots in the upper panels. This region can be defined either based on an LD cut-off or based on a simple zoom in. In addition, to facilitate inspection, LocusTrack can display every assessed SNP in a track-like fashion which uses the same color-coding of SNPs in the first panel.

Finally, our application generates an R object with the annotations requested for each specified loci (e.g. genes located in that region, LD, and the information of the tracks selected), facilitating the GWAS annotation.

## Results and discussion

Figure 1 shows an example of a LocusTrack plot. We used the genome wide significant associated SNP rs548181 from the publicly available GWAS results of the PGC (Psychiatric Genomics Consortium) schizophrenia working group [8]. The upper panel of the plot shows the extent of regional LD in the CEU population, the SNP p-values and the genes contained within the region. In addition, unlike similar applications such as LocusZoom [4] our software provides users with the option of displaying genomic tracks in the lower panel, which greatly facilitates the visual inspection of genomic context and annotation. In our example, the information in the lower panel corresponds to a region 5X zoom into the upper panel region. Further, the conservation extent within the region, as well as 3 different annotation tracks illustrating regulatory elements (i.e. transcription factor binding site clusters, DNAseI clusters and inferred chromatin state in human embryonic stem cells) are shown. We also include an LD track that shows the LD pattern in a different population (i.e. Han Chinese from Beijing; CHB). Our plot shows that the region around SNP rs548181 contains a probable transcription factor binding site and a DNaseI hot spot. Finally, the computationally inferred chromatin states by the ENCODE/Broad indicates that the SNP is within a putative active promoter region in human embryonic stem cells (hESC). This information may be useful for functional annotation and support hypothesis generation toward the follow up of GWAS hits.

## Conclusions

We created a simple and intuitive application that allows the user to easily generate regional plots of GWAS results that incorporate both custom genomic annotations and tracks produced by the ENCODE project [9] and available from the UCSC genome browser [10]. LocusTrack facilitates visual inspection and annotation of genomic elements neighbouring associated loci. Our web application offers an easy way to handle GWAS and annotation files and adds functionality to popular existing tools LocusZoom and SNAP-plot.

### Availability

LocusTrack was written in R and runs within an R script wrapper. It implements R core graphics to generate the figures and the Bioconductor package rtracklayer [7] to extract UCSC tracks. Recombination data was downloaded from http://ebi.edu.au/ftp/software/software/ensembl/encode/users/anshul/temp/chromatinVariation/rawdata/phasing/geneticMaps/ and compressed into an R object for quicker access. Pairwise linkage disequilibrium is computed using PLINK (1.9) [11] and the 1000 Genomes Project [12] data (23/11/2010 version).

The web application allows for an easy file management. Users can upload GWAS results files with data organised in columns with SNPs, positions and P-values as well as annotation tracks to the web server. The time needed to generate a single plot depends on the number of tracks selected, the size of the region to be displayed, and number of jobs currently running on the server. However, it is generally within a few minutes.

Finally, although LocusTrack is mainly intended as a web application, it is possible to run it locally on any Unix machine. The user only requires to have R and PLINK installed and to download the LocusTrack scripts along with the associated data from http://gump.qimr.edu.au/general/gabrieC/LocusTrack/downloads.html.

Documentation of all features and the scripts are available on the LocusTrack website.
